# Supplementary material for: Essential Trace Elements Status in Portuguese Pregnant Women and Their Association with Maternal and Neonatal Outcomes: A Prospective Study from the IoMum Cohort
Source: Biology (Basel). 2023 Oct 21;12(10):1351. doi: 10.3390/biology12101351 (PMC10604833; doi:10.3390/biology12101351)
Supplement: Supplementary file 1 [file biology-12-01351-s001.zip › biology-2557325-supplementary.pdf]

biology

MDPI

Table S1. Multimineral and vitamin content of supplements reported to be used by participants.

	RDD	Omega-3 (DHA/EPA) (mg)	Vitamins													Minerals												
			B6 (mg)	B5 (mg)	B3 (mg)	B2 (mg)	B1 (mg)	B8 e B7 (mg)	B9 (µg)	B12 (µg)	C (mg)	A (µg)	D (µg)	E (mg)	K (µg)	I (µg)	Fe (mg)	Zn (mg)	Mg (mg)	Cu (µg)	P (mg)	Mn (mg)	K (µg)	Ca (mg)	Mo (µg)	Cr (µg)	Se (µg)	Co (µg)
MV/MMF 1	1 cap	200	2	3	16	1.6	0.7	75	400	2	30	300	10	5	35	150	30	7	0	600	0	0	0	0	0	0	55	0
MV/MMF 2	1 pill	0	2.1	6	16	2.1	1.8	50	300	3	80	800	5	12	30	100	0	5	0	500	124	2	0	160	0	0	30	0
MV/MMF 3	1 cap	200	1.4	6	16	1.4	1.1	50	400	2.5	40	0	10	0	10	200	28	10	0	0	0	0	0	0	0	0	55	0
MV/MMF 4	1 cap	260 (31.2/46.8)	4	6	18	1.6	1.4	150	500	2.5	60	0	0	22.38	0	200	28	3.75	166	500	0	1	134	0	0	0	55	0
MV/MMF 5	1 pill	0	0	0	0	0	0	0	400	2	0	0	0	0	0	0	0	0	0	0	0	0	0	0	0	0	0	0
MV/MMF 6	14 g of powder + 3 caps	360 (300/40)	5	18	36	3.5	3	150	500	9	110	2000	5	50	60	150	5	5	180	1000	0	0	0	400	80	60	30	0
MV/MMF 7	1 pill	0	10	6	20	5	8	150	400	20	90	0	20	0	0	150	14	15	60	1000	0	0	0	0	0	0	50	0
MV/MMF 8	1 pill	0	10	6		2	3	150	400	6	70	0	10	0	70	150	17	15	150	1000	0	0	0	0	0	0	30	0
MV/MMF 9	1 pill	0	2.4	0	20	1.7	1.4	0	400	6	60	1500	10	0	0	150	18	0	100	0	0	0	0	125	0	0	0	0
MV/MMF 10	2 pills	0	1.25	5	10	1	0.85	150	400	4	50	1800	5	10	0	75	14	11.5	227	1000	0	0.5	0	652	0	12.5	12.5	0
MV/MMF 11	1 pill	0	0	0	0	0	0	0	350	0	0	0	0	0	0	0	100	0	0	0	0	0	0	0	0	0	0	0
MV/MMF 12	1 cap	100	1.4	0	0	0	0	0	500	2.5	80	800	5	12	0	200	30	0	0	0	0	0	0	200	0	0	20	0
MV/MMF 13	1 pill	0	0	0	0	0	0	0	1000	0	0	0	0	0	0	0	90	0	0	0	0	0	0	0	0	0	0	0
MV/MMF 14	1 cap + 1 pill	477 (300/33)	1.4	6	16	1.4	1.1	50	400	2.5	20	0	0	12	0	200	28	10	0	0	0	0	61.6	0	0	0	0	0
MV/MMF 15	1 pill	0	0	0	0	0	0	0	400	2	0	0	0	0	0	200	0	0	0	0	0	0	0	0	0	0	0	0
MV/MMF 16	1 cap	100	1.4	0	0	0	0	0	500	2.5	80	800	5	12	0	200	30	0	0	0	0	0	0	200	0	0	20	0
MV/MMF 17	1 cap	46	1.4	0	16	1.4	1.1	50	500	2.6	0	0	5	0	0	0	30	0	0	1000	0	2	0	0	0	0	60	0
MV/MMF 18	1 cap	250	1.4	6	16	1.4	1.1	50	400	2.5	80	0	5	12	0	200	14	10	45	500	18	1	0	24	0	0	0	0
MV/MMF 19	2 sachets	0	0.7	0	0	0	0	0	400	0	0	0	10	0	0	0	0	5	0	0	0	2	0	0	0	0	0	0
MV/MMF 20	1 cap	0	0	0	0	0	0	0	400	0	0	0	0	0	0	200	0	0	0	0	0	0	0	0	0	0	0	0
MV/MMF 21	1 cap	240 (200/40)	2	6	18	1.6	1.4	150	500	2.6	80	0	5	15	0	200	28	10	100	1000	0	1.09	0	0	0	0	60	0

MV/MMF 22	1 cap	318 (200/22)	2	6	18	1.6	1.5	150	500	2.6	80	0	5	15	0	200	28	10	100	1000	0	1.09	0	0	0	0	62.6	0
SIF 1	1 pill	0	0	0	0	0	0	0	500	0	0	0	0	0	0	0	0	0	0	0	0	0	0	0	0	0	0	0
SIF 2	1 pill	0	0	0	0	0	0	0	500	0	0	0	0	0	0	0	0	0	0	0	0	0	0	0	0	0	0	0
SIF 3	1 pill	0	0	0	0	0	0	0	400	0	0	0	0	0	0	0	0	0	0	0	0	0	0	0	0	0	0	0
SIF 4	1 pill	0	0	0	0	0	0	0	500	0	0	0	0	0	0	0	0	0	0	0	0	0	0	0	0	0	0	0
SIF 5	1 pill	0	0	0	0	0	0	0	0	0	0	0	0	0	0	200	0	0	0	0	0	0	0	0	0	0	0	0

Legend: Cap, capsule; Ca, calcium; Co, cobalt; Cr, chromium; Cu, copper; DHA, docosahexaenoic acid; EPA, eicosapentaenoic acid; Fe, iron; I, iodine; K, potassium; Mg, magnesium; Mn, manganese; Mo, molybdenum; MV/MMF, multivitamin/multimineral formulation; P, phosphorus; RDD, recommended daily dose; Se, selenium; SIF, single ingredient formulation; Vit, vitamin; Zn, zinc.

Table S2. Calculated percentiles for Zinc and Manganese urinary concentrations.

		ETEs (μg/L)	
		Mn	Zn
Percentiles	5th	0.14	53.0
	10th	0.30	82.8
	15th	0.45	104.0
	20th	0.58	121.7
	25th	0.71	145.8
	30th	0.86	163.6
	35th	1.02	183.9
	40th	1.22	209.2
	45th	1.42	234.8
	50th	1.67	256.9
	55th	1.89	282.7
	60th	2.11	317.6
	65th	2.46	353.1
	70th	2.70	410.2
	75th	3.05	446.7
	80th	3.55	501.9
	85th	4.07	550.1
	90th	4.85	638.5
	95th	5.95	839.3

Legend: ETEs, essential trace elements; Mn, manganese; Zn, zinc.

Table S3. Sociodemographic characteristics of pregnant women by urinary levels of ETEs.

Characteristics	n	%	Co (µg/g)			Cu (µg/g)			Mn (µg/g)			Mo (µg/g)			Zn (µg/g)		
			Median	(P25; P75)	*p*	Median	(P25; P75)	*p*	Median	(P25; P75)	*p*	Median	(P25; P75)	*p*	Median	(P25; P75)	*p*
Sub-cohort																	
Porto	337	53	0.28	(0.13; 0.49)	<0.001[a]	18.98	(11.50; 35.2)	<0.001[a]	3.59	(2.04; 5.54)	<0.001[a]	40.56	(28.7; 57.3)	<0.001[a]	310.33	(201.0; 497.3)	0.045[a]
Lisbon	298	47	0.39	(0.28; 0.61)		10.37	(8.20; 15.1)		0.92	(0.49; 1.79)		53.03	(39.2; 70.0)		360.57	(239.9; 510.9)	
Education level																	
Low (≤ 9 years)	155	25	0.29	(0.15; 0.47)		19.28	(11.2; 32.3)		3.50	(1.86; 5.46)		38.67	(27.2; 55.2)		292.34	(187.2; 474.5)	
Medium (10 to 12 years)	181	29	0.34	(0.15; 0.65)	<0.001[b]	14.68	(9.90; 24.7)	<0.001[b]	2.45	(1.33; 4.69)	<0.001[b]	44.94	(31.8; 56.4)	<0.001[b]	377.39	(245.9; 534.0)	0.016[b]
University (≥ 13 years)	282	46	0.37	(0.25; 0.57)		10.96	(8.4;0 16.1)		1.00	(0.53; 2.21)		53.62	(38.7; 70.8)		339.51	(223.9; 510.2)	
Smoking habits																	
Non-smoker	443	70	0.33	(0.18; 0.54)		13.24	(9.00; 22.9)		1.85	(0.82; 3.77)		47.82	(33.7; 64.9)		328.03	(215.8; 504.4)	
Former smoker	132	21	0.37	(0.24; 0.69)	0.025[b]	13.55	(8.80; 22.4)	0.598[b]	1.82	(0.91; 3.94)	0.015[b]	51.49	(36.4; 65.0)	0.012[b]	337.37	(241.7; 517.0)	0.473[b]
Smoker	56	9	0.35	(0.26; 0.51)		14.99	(9.40; 23.6)		3.46	(1.19; 5.10)		39.05	(30.3; 54.5)		321.79	(229.6; 484.4)	
Pre-pregnancy BMI																	
Underweight	35	6	0.32	(0.15; 0.68)		13.65	(10.0; 17.4)		2.54	(1.27; 4.28)		51.44	(33.7; 69.9)		376.25	(236.8; 568.0)	
Normal weight	400	63	0.36	(0.22; 0.59)	0.058[b]	13.57	(9.10; 23.9)	0.940[b]	2.04	(0.90; 4.25)	0.236[b]	49.76	(35.9; 68.2)	<0.001[b]	325.95	(223.3; 507.6)	0.829[b]
Overweight	114	18	0.31	(0.17; 0.49)		13.66	(9.50; 21.2)		1.93	(0.80; 3.24)		44.36	(32.0; 56.8)		339.12	(230.0; 497.3)	
Obese	85	13	0.30	(0.17; 0.46)		13.80	(8.50; 24.4)		1.72	(0.78; 3.47)		38.45	(30.5; 53.2)		314.97	(200.6; 518.2)	
1st trimester weight variation																	
Below adequacy	132	21	0.32	(0.17; 0.48)		14.47	(9.60; 27.6)		1.79	(0.85; 4.04)		44.45	(32.6; 61.9)		343.24	(239.7; 517.6)	
Adequate	189	31	0.34	(0.20; 0.55)	0.093[b]	13.49	(9.30; 21.8)	0.292[b]	2.18	(0.90; 4.03)	0.729[b]	49.35	(37.2; 67.3)	0.145[b]	326.37	(236.4; 517.3)	0.347[b]
Above adequacy	293	48	0.36	(0.21; 0.58)		12.93	(8.80; 21.1)		1.87	(0.89; 4.08)		47.86	(33.1; 63.1)		322.37	(199.2; 491.1)	

Legend: Co, cobalt; Cu, copper; ETEs, essential trace elements; Mn, manganese; Mo, molybdenum; Zn, zinc.
[a]Mann-Whitney.
[b]Kruskal-Wallis.

Table S4. Urinary concentrations of ETEs in the 1st trimester by frequency of intake of dairy products, eggs, fish, seafood, offal, leguminous and nuts.

Food intake	n	%	Co (μg/g)			Cu (μg/g)			Mn (μg/g)			Mo (μg/g)			Zn (μg/g)		
			Median	(P25; P75)	*p*	Median	(P25; P75)	*p*	Median	(P25; P75)	*p*	Median	(P25; P75)	*p*	Median	(P25; P75)	*p*
Milk																	
< 3 times a month	178	28.4	0.33	(0.19; 0.58)		13.57	(8.98; 23.9)		1.76	(0.65; 3.73)		49.24	(34.5; 68.2)		382.52	(263.1; 529.8)	
1 a 6 times a week	157	25.0	0.32	(0.19; 0.54)	0.610[a]	12.51	(8.90; 21.5)	0.437[a]	1.80	(0.91; 3.71)	0.019[a]	47.96	(34.2; 61.5)	0.741[a]	304.30	(202.1; 476.7)	0.039[a]
1 time a day	216	34.4	0.36	(0.20; 0.54)		13.94	(9.00; 23.5)		2.17	(0.92; 4.03)		47.23	(34.2; 62.0)		317.20	(215.3; 479.2)	
≥ 2 times a day	76	12.1	0.37	(0.20; 0.59)		14.52	(9.69; 23.2)		2.40	(1.12; 4.98)		44.73	(30.7; 63.4)		322.43	(211.3; 585.5)	
Yoghurt																	
< 3 times a month	106	16.9	0.34	(0.21; 0.54)		13.50	(9.42; 23.1)		1.83	(0.65; 3.99)		46.33	(32.4; 65.4)		340.57	(259.1; 498.6)	
1 a 6 times a week	274	43.7	0.35	(0.20; 0.55)	0.841[a]	12.47	(8.72; 20.6)	0.024[a]	1.76	(0.80; 3.45)	0.015[a]	46.46	(34.4; 62.0)	0.792[a]	322.52	(203.8; 498.2)	0.630[a]
1 time a day	182	29.0	0.35	(0.19; 0.59)		14.46	(8.89; 25.6)		2.15	(0.91; 4.75)		48.89	(34.4; 67.2)		354.57	(228.8; 539.9)	
≥ 2 times a day	65	10.4	0.36	(0.16; 0.53)		15.74	(10.4; 26.4)		2.78	(1.16; 4.97)		48.22	(33.5; 63.3)		322.37	(207.9; 528.7)	
Cheese																	
< 3 times a month	97	15.5	0.35	(0.20; 0.53)		13.24	(8.47; 23.9)		1.69	(0.84; 3.45)		48.22	(32.4; 64.4)		348.56	(231.4; 559.8)	
1 a 6 times a week	341	54.5	0.34	(0.20; 0.57)	0.453[a]	13.89	(9.52; 22.7)	0.008[a]	2.13	(0.92; 4.07)	0.196[a]	47.37	(34.2; 62.0)	0.692[a]	326.37	(228.5; 515.2)	0.612[a]
1 time a day	140	22.4	0.33	(0.18; 0.53)		11.58	(8.20; 19.0)		1.80	(0.73; 3.55)		47.26	(35.2; 66.2)		316.58	(206.6; 475.0)	
≥ 2 times a day	48	7.7	0.39	(0.26; 0.62)		17.32	(10.87; 26.9)		2.61	(1.29; 4.28)		52.92	(35.6; 64.4)		382.13	(233.9; 512.5)	
Eggs																	
< 3 times a month	126	20.2	0.32	(0.19; 0.53)		14.53	(8.91; 23.0)		2.56	(1.08; 4.75)		44.67	(32.1; 58.3)		330.46	(232.9; 498.6)	
1 a 3 times a week	410	65.6	0.35	(0.19; 0.55)	0.380[a]	13.68	(9.35; 23.6)	0.271[a]	1.93	(0.89; 3.99)	0.004[a]	48.17	(34.4; 64.9)	0.100[a]	331.24	(220.1; 505.6)	0.853[a]
≥ 4 times a week	89	14.2	0.38	(0.23; 0.59)		12.42	(8.60; 18.1)		1.37	(0.61; 2.77)		49.80	(37.1; 69.8)		361.72	(232.9; 542.4)	
Fish																	
< 3 times a month	71	11.6	0.34	(0.17; 0.54)		14.62	(8.72; 25.6)		2.31	(0.87; 4.17)		42.68	(32.1; 59.3)		376.31	(240.7; 491.5)	
1 a 3 times a week	407	66.6	0.34	(0.19; 0.57)	0.555[a]	13.42	(9.46; 21.7)	0.266[a]	1.98	(0.90; 4.07)	0.011[a]	47.96	(34.2; 63.5)	0.129[a]	326.37	(216.5; 515.2)	0.755[a]
≥ 4 times a week	133	21.8	0.38	(0.24; 0.55)		12.48	(8.38; 20.6)		1.46	(0.71; 2.96)		50.65	(34.7; 66.1)		321.39	(236.9; 476.8)	
Seafood																	
do not eat Seafood at all	180	60.8	0.39	(0.28; 0.62)	0.933[b]	10.39	(8.20; 15.2)	0.980[b]	0.92	(0.49; 1.82)	0.413[b]	53.40	(41.5; 74.5)	0.092[b]	363.32	(262.1; 510.6)	0.294[b]
≥ 1 time a month	116	39.2	0.39	(0.27; 0.61)		10.36	(8.33; 15.1)		0.91	(0.49; 1.52)		51.18	(38.3; 65.3)		331.08	(222.0; 528.9)	
Offal																	

do not eat Offal at all	228	80.0	0.40	(0.28; 0.61)	0.400[b]	10.39	(8.23; 14.9)	0.901[b]	0.93	(0.50; 1.82)	0.854[b]	53.34	(40.3; 70.1)	0.323[b]	361.60	(249.0; 510.6)	0.950[b]
≥ 1 time a month	57	20.0	0.33	(0.26; 0.60)		10.24	(8.22; 15.1)		0.93	(0.48; 1.75)		51.46	(36.4; 67.9)		372.57	(225.7; 556.6)	
Leguminous																	
< 3 times a month	58	20.4	0.40	(0.25; 0.57)		9.36	(7.82; 13.1)		0.73	(0.46; 1.68)		44.89	(34.9; 59.0)		339.12	(242.8; 463.5)	
1 a 3 times a week	160	56.3	0.38	(0.29; 0.61)	0.713[a]	10.78	(8.27; 15.3)	0.158[a]	0.94	(0.49; 2.04)	0.257[a]	53.47	(40.3; 71.1)	0.003[a]	352.32	(245.0; 521.7)	0.773[a]
≥ 4 times a week	66	23.2	0.42	(0.27; 0.67)		10.40	(8.78; 15.3)		1.11	(0.59; 1.70)		56.13	(46.9; 76.7)		375.61	(237.4; 550.7)	
Nuts																	
< 3 times a month	82	29.0	0.38	(0.28; 0.59)		9.44	(7.82; 13.4)		0.80	(0.47; 1.46)		52.18	(41.8; 77.4)		372.10	(239.9; 498.6)	
1 a 3 times a week	108	38.2	0.39	(0.28; 0.60)	0.903[a]	10.84	(8.33; 14.9)	0.074[a]	0.94	(0.49; 1.83)	0.274[a]	49.49	(37.0; 65.5)	0.151[a]	340.57	(223.8; 516.0)	0.573[a]
≥ 4 times a week	93	32.9	0.41	(0.27; 0.64)		11.39	(8.78; 16.0)		1.07	(0.51; 2.15)		54.59	(42.7; 71.7)		373.83	(268.5; 550.7)	

Legend: Co, cobalt; Cu, copper; ETEs, essential trace elements; Mn, manganese; Mo, molybdenum; Zn, zinc.
[a]Kruskal-Wallis.
[b]Mann-Whitney.

Table S5. Servings of food groups considered in the questionnaire.

Food group	Serving
Milk	1 cup (250 ml)
Yogurt	1 solid yogurt (150 g) or liquid yogurt (180 ml)
Cheese	2 slices of cheese (40 g)
Eggs	1 egg (56 g)
Fish	1 fish fillet (125 g)
Seafood	Not specified
Offal	¼ of a plate (90 g)
Leguminous	3 tablespoons (80 g)
Nuts	1 full hand (25 g)

Table S6. Association between first trimester urinary ETEs levels and pregnancy complications.

ETEs (µg/g)	Uncomplicated pregnancy (n = 476)			Pregnancy complications (n = 120)			*p*[a]
	Median	P25	P75	Median	P25	P75	
Co	0.35	0.21	0.57	0.31	0.19	0.53	0.162
Cu	13.7	8.98	24.0	12.4	8.8	18.0	0.080
Mn	1.92	0.85	4.05	1.53	0.70	3.44	0.052
Mo	47.7	33.9	63.3	48.5	34.5	64.9	0.900
Zn	327.1	216.4	507.9	365.1	240.0	548.5	0.199

Legend: Co, cobalt; Cu, copper; ETEs, essential trace elements; Mn, manganese; Mo, molybdenum; Zn, zinc.
[a]Mann-Whitney.

Table S7. First-trimester maternal urinary Mn and Zn concentrations according to the type of pregnancy complication.

Type of pregnancy complications		n	%	Mn (µg/g)			Zn (µg/g)		
				Median	(P25; P75)	*p*[a]	Median	(P25; P75)	*p*[a]
PE									
	No	579	97.3	1.85	(0.84; 3.94)	0.007	334.47	(220.2; 513.2)	0.377
	Yes	16	2.7	0.87	(0.44; 1.56)		357.48	(281; 563.5)	
PTB									
	No	571	95.8	1.82	(0.78; 3.86)	0.261	338.97	(224.1; 513.2)	0.493
	Yes	25	4.2	2.17	(1.08; 4.08)		288.52	(146.2; 548.4)	

Legend: Co, cobalt; Cu, copper; ETEs, essential trace elements; Mn, manganese; Mo, molybdenum; PE, preeclampsia; PTB, preterm birth; Zn, zinc.
[a]Mann-Whitney.

Table S8. Association between first trimester urinary ETEs levels and neonatal outcomes.

Neonatal outcomes	n	%	Co (µg/g)			Cu (µg/g)			Mn (µg/g)			Mo (µg/g)			Zn (µg/g)		
			Median	(P25; P75)	*p*	Median	(P25; P75)	*p*	Median	(P25; P75)	*p*	Median	(P25; P75)	*p*	Median	(P25; P75)	*p*
Birth weight adequacy																	
SGA	34	5	0.29	(0.15; 0.50)		13.85	(9.35; 21.1)		3.22	(1.36; 4.34)		49.45	(36.4; 65.6)		301.58	(173.8; 382.5)	
AGA	580	91	0.35	(0.20; 0.57)	0.099[a]	13.68	(9.01; 23.1)	0.863[a]	1.92	(0.86; 3.95)	0.096[a]	47.06	(33.3; 63.2)	0.807[a]	334.48	(225.7; 521.0)	0.132[a]
LGA	20	3	0.30	(0.13; 0.44)		12.32	(10.0; 15.6)		1.68	(0.72; 2.97)		50.46	(40.7; 63.5)		375.21	(272.0; 461.1)	
Birth head circumference adequacy																	
SGA	32	5	0.27	(0.14; 0.42)		14.30	(9.12; 17.8)		2.79	(1.06; 4.41)		47.85	(27.0; 64.9)		252.26	(166.2; 422.3)	
AGA	523	86	0.35	(0.20; 0.57)	0.117[a]	13.66	(8.98; 23.1)	0.451[a]	2.05	(0.86; 3.96)	0.042[a]	47.10	(33.6; 63.5)	0.520[a]	341.90	(223.9; 513.2)	0.112[a]
LGA	56	9	0.29	(0.18; 0.50)		12.08	(9.25; 20.6)		1.43	(0.71; 2.70)		49.46	(35.7; 61.2)		364.84	(262.7; 559.2)	
Birth length adequacy																	
SGA	49	8	0.33	(0.21; 0.48)		13.50	(8.97; 17.1)		1.82	(0.74; 3.53)		48.22	(34.5; 61.8)		299.88	(250.4; 465.6)	
AGA	570	91	0.35	(0.19; 0.57)	0.676[b]	13.65	(9.12; 23.2)	0.301[b]	2.05	(0.87; 4.07)	0.376[b]	47.57	(33.5; 63.4)	0.857[b]	342.04	(220.2; 517.1)	0.716[b]
LGA	4	1	nd	nd		nd	nd		nd	nd		nd	nd		nd	nd	

Legend: AGA, adequate for gestational age; ETEs, essential trace elements; LGA, large for gestational age; nd, not determined due to small sample size; SGA, small for gestational age.

[a]Kruskal-Wallis.

[b]Mann-Whitney.

Table S9. Urinary cobalt, copper, manganese, molybdenum and zinc concentrations, as median or mean, among pregnant women and general adult population reported in our cohort and in literature.

ETEs	ETEs concentrations (μg/L) IoMum (1st Trimester)	Pregnant women (1st Trimester)	General population
Cobalt	0.31[a]	0.40[a41]	0.18[a44]
		0.42[a42]	0.30[a45]
			0.39[b46]
			0.70[a47]
Copper	11.1[a]	15.0[a41]	8.18[a44]
			6.90[a45]
			9.00[b46]
			14.9[a46]
Manganese	1.67[a]	0.20[a41]	< 0.04[a44]
			0.31[a45]
			0.09[b46]
			0.16[a46]
Molybdenum	39.3[a]	38.5[a43]	31.3[a44]
			20.0[a45]
			38.0[b46]
Zinc	256.9[a]	290.0[a41]	256.0[a44]
			195.0[a45]
			269.0[b46]

Legend: ETEs, essential trace elements.
[a]Median.
[b]Mean.

Table S10. Maternal urinary concentrations of Co, Cu, Mn and Zn in the 1st trimester according to food supplement use.

Supplementation	n	%	Co (µg/g)			Cu (µg/g)			Mn (µg/g)			Zn (µg/g)		
			Median	(P25; P75)	p^a	Median	(P25; P75)	p^a	Median	(P25; P75)	p^a	Median	(P25; P75)	p^a
NS	23	4.3	0.38	(0.29; 0.68)	0.707	19.28	(11.8; 34.9)	0.004	2.75	(1.93; 4.98)	<0.001	299.88	(215.8; 603)	0.550
MV/MMF	249	46.0	0.38	(0.24; 0.58)		11.59	(8.38; 19.3)		1.38	(0.61; 2.82)		374.44	(239.9; 548.4)	

Legend: Co, cobalt; Cu, copper; Mn, manganese; Mo, molybdenum; MV/MMF, multivitamin/multimineral formulation users; NS, non-users of supplements; Zn, zinc.
[a]Mann-Whitney.
